# Using Bumble Bee Watch to investigate the accuracy and perception of bumble bee (*Bombus* spp.) identification by community scientists

**DOI:** 10.7717/peerj.9412

**Published:** 2020-06-29

**Authors:** Victoria J. MacPhail, Shelby D. Gibson, Richard Hatfield, Sheila R. Colla

**Affiliations:** 1 Faculty of Environmental Studies, York University, Toronto, ON, Canada; 2 Department of Biology, York University, Toronto, ON, Canada; 3 The Xerces Society for Invertebrate Conservation, Portland, OR, USA

**Keywords:** Citizen science, Community science, Citizen scientist, Bumble bees, *Bombus*, Identification, Accuracy, Perception of ease of identification, Bumble Bee Watch, Expert verification

## Abstract

Community science programs provide an opportunity to gather scientific data to inform conservation policy and management. This study examines the accuracy of community science identifications submitted to the North American Bumble Bee Watch program on a per species level and as compared to each species’ conservation status, as well as users (members of the public) and experts (those with expertise in the field of bumble bee biology) perceived ease of species identification. Photos of bumble bees (Hymenoptera: Apidae: *Bombus*) are submitted to the program by users and verified (species name corrected or assigned as necessary) by an expert. Over 22,000 records from over 4,900 users were used in the analyses. Accuracy was measured in two ways: percent agreement (percent of all records submitted correctly by users) and veracity (percent of all verified records submitted correctly by the users). Users generally perceived it harder to identify species than experts. User perceptions were not significantly different from the observed percent agreement or veracity, while expert perceptions were significantly different (overly optimistic) from the observed percent agreement but not the veracity. We compared user submitted names to final expert verified names and found that, for all species combined, the average percent agreement was 53.20% while the average veracity was 55.86%. There was a wide range in percent agreement values per species, although sample size and the role of chance did affect some species agreements. As the conservation status of species increased to higher levels of extinction risk, species were increasingly more likely to have a lower percent agreement but higher levels of veracity than species of least concern. For each species name submitted, the number of different species verified by experts varied from 1 to 32. Future research may investigate which factors relate to success in user identification through community science. These findings could play a role in informing the design of community science programs in the future, including for use in long-term and national-level monitoring of wild pollinators.

## Introduction

Community science, which is also known as citizen science, is a popular tool in conservation biology that involves public participation in scientific data collection (Silvertown, 2009; Crall et al., 2011; Kremen, Ullmann & Thorp, 2011; Lye et al., 2011; Dickinson et al., 2012; Lebuhn et al., 2012; Roy et al., 2012; Birkin & Goulson, 2015; Kobori et al., 2016; Kosmala et al., 2016; McKinley et al., 2017). The main strengths of community science include increasing the spatial and temporal scale and magnitude of sampling efforts; reducing the cost of sampling; and creating educational and recreational benefits for participants (Bonney et al., 2009; Kremen, Ullmann & Thorp, 2011; Dickinson et al., 2012; Lebuhn et al., 2012; Birkin & Goulson, 2015; McKinley et al., 2017; Falk et al., 2019).

Accurate species level identification is an important first step in conservation and is an essential pre-requisite for effective conservation management decisions (Le Féon et al., 2011; Kremen, Ullmann & Thorp, 2011). Previous research has found that community scientist data, when unreviewed or unverified by experts, can contain errors that significantly influence the interpretation of results by experts (Gardiner et al., 2012; Comont & Ashbrook, 2017). Problematic errors include the overestimation of rare or at-risk species, as well as underestimation of common species, inflated species richness and significant increases in species diversity (Dickinson, Zuckerberg & Bonter, 2010; Gardiner et al., 2012; Silvertown et al., 2013; Comont & Ashbrook, 2017; Falk et al., 2019).

Verification is part of expert assisted community science programs where experts in the field review a submission to determine if the identification is correct or incorrect, which can increase data quality (Le Féon et al., 2011; Gardiner et al., 2012; Comont & Ashbrook, 2017; Falk et al., 2019). As such, expert assisted community science projects have a higher scientific value, particularly for conservation management decisions (Dickinson et al., 2012; Crall et al., 2011; Gardiner et al., 2012; Falk et al., 2019).

Bumble bees (Hymenoptera: Apidae: *Bombus*) are native throughout the Northern Hemisphere and in South America with their diversity centered in temperate and mountainous regions (Williams et al., 2014). They are important pollinators in natural and agro-ecosystems (Corbet, Williams & Osborne, 1991; Buchmann & Nabhan, 1996). A portion of North America’s bumble bee species have been found to be in decline in recent years (Colla & Packer, 2008; Grixti et al., 2009; Cameron et al., 2011; IUCN Red List of Threatened Species (IUCN), 2019) (see Table 1). There are several factors that have been identified as drivers of bumble bee decline, including: pathogen transmission from managed bees, habitat loss, pesticides, interspecific competition with non-native species and climate change (Colla et al., 2006; Otterstatter & Thomson, 2008; Cameron et al., 2011; Szabo et al., 2012; Goulson et al., 2015; Kerr et al., 2015; McMahon et al., 2015; Papanikolaou et al., 2017; Cameron & Sadd, 2020; Soroye, Newbold & Kerr, 2020). As *Bombus* is a genus of relatively large bees, sometimes with distinct physical features, experts are often able to provide a confident identification to species level from photos, although it does depend on the species and angle and quality of the photo (Lye et al., 2011; Suzuki-Ohno et al., 2017; Falk et al., 2019).

**Table 1:** The common and scientific names and taxonomic authorities for the 46 species of bumble bees found in North America as well as their IUCN Red List extinction risk status for their North American range.

Genus	Species	Authority	Common name	IUCN status
*Bombus*	*affinis*	Cresson, 1863	Rusty-Patched Bumble Bee	CR
*Bombus*	*appositus*	Cresson, 1878	White-Shouldered Bumble Bee	LC
*Bombus*	*auricomus*	(Robertson, 1903)	Black and Gold Bumble bee	LC
*Bombus*	*bifarius*	Cresson, 1878	Two Form Bumble Bee	LC
*Bombus*	*bimaculatus*	Cresson, 1863	Two-spotted Bumble Bee	LC
*Bombus*	*bohemicus*	Seidl, 1838	Ashton Cuckoo Bumble Bee	DD
*Bombus*	*borealis*	Kirby, 1837	Northern Amber Bumble Bee	LC
*Bombus*	*caliginosus*	(Frison, 1927)	Obscure Bumble Bee	VU
*Bombus*	*centralis*	Cresson, 1864	Central Bumble Bee	LC
*Bombus*	*citrinus*	(Smith, 1854)	Lemon Cuckoo Bumble Bee	LC
*Bombus*	*crotchii*	Cresson, 1878	Crotch Bumble Bee	EN
*Bombus*	*cryptarum*	(Fabricius, 1775)	Cryptic Bumble Bee	DD
*Bombus*	*distinguendus*	Morawitz, 1869	Northern Yellow Bumble Bee	DD
*Bombus*	*fervidus*	(Fabricius, 1798)	Yellow Bumble Bee	VU
*Bombus*	*flavidus*	Eversmann, 1852	Fernald Cuckoo Bumble Bee	DD
*Bombus*	*flavifrons*	Cresson, 1963	Yellow Head Bumble Bee	LC
*Bombus*	*franklini*	(Frison, 1921)	Franklin Bumble Bee	CR
*Bombus*	*fraternus*	(Smith, 1854)	Southern Plains Bumble Bee	EN
*Bombus*	*frigidus*	Smith, 1854	Frigid Bumble Bee	LC
*Bombus*	*griseocollis*	(DeGeer, 1773)	Brown-belted Bumble Bee	LC
*Bombus*	*huntii*	Greene, 1860	Hunt Bumble Bee	LC
*Bombus*	*hyperboreus*	Schönherr, 1809	High Arctic Bumble Bee	DD
*Bombus*	*impatiens*	Cresson, 1863	Common Eastern Bumble Bee	LC
*Bombus*	*insularis*	(Smith, 1861)	Indiscriminate Cuckoo Bumble Bee	LC
*Bombus*	*jonellus*	(Kirby, 1802)	White Tail Bumble Bee	DD
*Bombus*	*kirbiellus*	Curtis, 1835	High Country Bumble Bee (*B. balteatus* Dahlbom, 1832 in Williams et al. (2014))	DD
*Bombus*	*melanopygus*	Nylander, 1848	Black Tail Bumble Bee	LC
*Bombus*	*mixtus*	Cresson, 1878	Fuzzy-horned Bumble Bee	LC
*Bombus*	*morrisoni*	Cresson, 1878	Morrison Bumble Bee	VU
*Bombus*	*neoboreus*	Sladen, 1919	Active bumble bee	DD
*Bombus*	*nevadensis*	Cresson, 1874	Nevada Bumble Bee	LC
*Bombus*	*occidentalis*	Greene, 1858	Western Bumble Bee	VU
*Bombus*	*pensylvanicus*	(DeGeer, 1773)	American Bumble Bee	VU
*Bombus*	*perplexus*	Cresson, 1863	Confusing Bumble Bee	LC
*Bombus*	*polaris*	Curtis, 1835	Polar Bumble Bee	DD
*Bombus*	*rufocinctus*	Cresson, 1863	Red-belted Bumble Bee	LC
*Bombus*	*sandersoni*	Franklin, 1913	Sanderson Bumble Bee	LC
*Bombus*	*sitkensis*	Nylander, 1848	Sitka bumble bee	LC
*Bombus*	*suckleyi*	Greene, 1860	Suckley Cuckoo Bumble Bee	CR
*Bombus*	*sylvicola*	Kirby, 1837	Forest Bumble Bee	LC
*Bombus*	*ternarius*	Say, 1837	Tri-colored Bumble Bee	LC
*Bombus*	*terricola*	Kirby, 1837	Yellow-banded Bumble Bee	VU
*Bombus*	*vagans*	Smith, 1854	Half-black Bumble Bee	LC
*Bombus*	*vandykei*	(Frison, 1927)	Van Dyke Bumble Bee	LC
*Bombus*	*variabilis*	Cresson, 1872	Variable Cuckoo Bumble Bee	CR
*Bombus*	*vosnesenskii*	Radoszkowski, 1862	Vosnesensky Bumble Bee	LC

**Note:**

Names and authorities from Williams et al. (2014). IUCN status abbreviations as per a July 30, 2019 export (IUCN Red List of Threatened Species (IUCN), 2019) and are as follows: DD, Data Deficient; LC, Least Concern; VU, Vulnerable; EN, Endangered; CR, Critically Endangered.

The community science program, Bumble Bee Watch, was launched as a website (www.bumblebeewatch.org) in March 2014. An iOS app (computer program for mobile devices such as phones and tablets) was added in July 2017, and an Android app was added in July 2018. Users (members of the public who participate in this program) submit photo observations of bumble bees with known dates and geographic locations throughout Canada and the United States. Users are not required to have any prior experience with or training in bumble bee identification to participate in the program, and can range in age, ability and location (MacPhail, Gibson & Colla, 2020). Photo documentation reduces the need for traditional monitoring protocols and equipment, including physical collection and curation of collected specimens (Silvertown, 2009; Lye et al., 2011; Lebuhn et al., 2012; Kobori et al., 2016), although photos are not always sufficient to identify some bumble bee species due to either the photo quality or the physical features of the specimen that need to be examined (Lye et al., 2011; Suzuki-Ohno et al., 2017; Falk et al., 2019).

Bumble Bee Watch is an example of expert assisted community science, where submissions from community scientists are reviewed by an expert in the field before entry into a species database (Le Féon et al., 2011; Gardiner et al., 2012). For Bumble Bee Watch, experts (aka verifiers) are individuals with significant expertise in the identification of bumble bees. Verifiers currently include some of the top bumble bee experts in North America, and all new verifiers must be referred to and vouched for by experts known to the current administrators (e.g., RH of the Xerces Society, SC of York University).

Community science monitoring projects benefit from accurate identifications by participants, which reduces verification time from experts, speeding up feedback to participants and enhancing educational value. Yet accuracy can vary amongst participants and programs. Examining accuracy in the Bumble Bee Watch program can be useful in assessing the over- and/or under-reporting of various bumble bee species compared to researcher collected data. These bumble bee records are highly valuable to conservation biologists who may use data for common species as a comparative background for looking at the status of at-risk species.

Assessing the accuracy of initial identification of both common (least concern) and at-risk (vulnerable through extinct) bumble bee species (as determined by the IUCN Red List of Threatened Species (IUCN) (2019)) is useful for Bumble Bee Watch to help assess the identification skill levels of participants, the need for expert verification, and the most common species misidentifications. Overall, a high accuracy of initial identification from users could suggest one or more of the following: (1) bumble bees are relatively easy to identify; (2) common bumble bee species are easy to identify; (3) experts and users make similar mistakes during identification; (4) users are taking advantage of the bumble bee identification materials provided by the program to have a high matching ability; (5) the identification key on the website and smart filter on the app is intuitive and easy to use; (6) users are experienced in bumble bee identification (e.g., enthusiasts, researchers, participants in targeted outreach programs); and/or (7) education and outreach programs are increasing the skill and abilities of users. Low accuracy of initial identification rates could suggest that: (1) bumble bees are hard to identify by novices and that expert assisted community science is required; (2) there is a need for program changes and/or additional training materials; (3) there are potential bias(es) by users; and/or (4) photographs are not clear enough or do not provide enough detail for verification. Accuracy of identification data can also be used to assess user interest and bias (Dickinson et al., 2012). Therefore, understanding what influences user accuracy could aid in the development of a long-term monitoring protocol for bumble bees in North America.

Effective long-term pollinator conservation requires collection of continuous, broad-scale data on native pollinator communities (Grixti et al., 2009; Cameron et al., 2011; Lebuhn et al., 2012; Goulson et al., 2015; Hatfield et al., 2015a, 2015b; Strange & Tripodi, 2019). Conservation biologists are dependent on accurate spatial and temporal data to determine the status and extinction risk of bumble bee species (Rodrigues et al., 2006; Mace et al., 2008; Cardoso et al., 2011; Colla et al., 2012; Hatfield et al., 2015a; Colla, 2016; MacPhail, Richardson & Colla, 2019). Since community science increases the ability for long-term monitoring programs to be developed, and community science data have recently been used to help assess many species (Howard & Davis, 2008; Kremen, Ullmann & Thorp, 2011; Lye et al., 2011; Suzuki-Ohno et al., 2017; United States Fish & Wildlife Service (USFWS), 2016a, 2016b, 2017a, 2017b, 2019a, 2019b, 2019c, 2019d; Committee on the Status of Endangered Wildlife in Canada (COSEWIC), 2018; MacPhail, Richardson & Colla, 2019), there is potential for Bumble Bee Watch data to contribute to long-term monitoring and management decisions for at-risk species. Community science data can also be used to monitor all species for trends in abundance, potentially providing an early warning before catastrophic collapses occur, such as have been seen in several North American bumble bees (Colla & Packer, 2008; Cameron et al., 2011; Colla et al., 2012; Bartomeus et al., 2013; MacPhail, Richardson & Colla, 2019; Mathiasson & Rehan, 2019; Richardson et al., 2019; Cameron & Sadd, 2020).

A recent meta-analysis showed that no significant difference, a correlation greater than 0.5, or a minimum percent agreement of 80% between community scientists and professional scientists is considered acceptable in terms of equivalency (Aceves-Bueno et al., 2017), although this does not take into account repercussions such as misidentification of specific species (such as those that are rare, endangered or invasive) (Austen et al., 2016; Stribling et al., 2008). Here, we use the reviewed database available from Bumble Bee Watch to assess the accuracy rate of data submitted by community scientists as assessed by bumble bee experts (The Xerces Society for Invertebrate Conservation et al., 2019).

Experts and users may have different perceptions on the ease or difficulty in identifying specific bumble bee species. Identifying differences in these perceptions could help those conducting outreach and training target their efforts and resources appropriately. To accomplish this, we completed a user and expert online survey to investigate the perceived ease of identification for each species and compared that to the actual observed accuracy in the program.

As the users who participate in the Bumble Bee Watch program have a range of bumble bee identification experience ranging from complete novice (majority of users) to expert (small number of users) (MacPhail, Gibson & Colla, 2020), there could be user bias or influence in the percent agreement between the user submitted name and expert verified one for some species. For example, if one user submitted most of the data for a specific species, and they are an individual with high identification accuracy, this could skew the percent agreement results as compared to species with identifications contributed by users with an average skill level. The reverse could also be true for a user with a lower than average identification accuracy.

We hypothesized (1) that there are differences in user identification accuracy amongst species; (2) that the agreement (relationship) between expert and user identifications is not due to chance alone; (3) that users have a higher accuracy of identification for common species than at-risk species; (4) that users and experts would have different perceptions of ease of identification; and (5) that the user perception of ease of identification would match their actual observed accuracy of identifications.

## Methods

### Data collection

As Bumble Bee Watch users submit photos of bumble bees, they are prompted to identify the bumble bee using a pictorial key filtered by location with choices amongst a group of options for various identification features (e.g., color-pattern). Submissions are marked as pending until they are reviewed by experts (those individuals determined by program administrators as having significant expertise in bumble bee identification) on Bumble Bee Watch, after which they are marked as verified (the expert confirms the species identification or corrects it as necessary), tentative (reviewed by an expert but uncertainty around the identification still exists) or invalid (identified by an expert to not be *Bombus*).

In this study, we compared the user submitted names to the final expert verified names. All nest and bee records were exported from Bumble Bee Watch on July 19, 2019 (The Xerces Society for Invertebrate Conservation et al., 2019). Records marked as pending and tentative were not included in any analyses of accuracy. Records marked as verified and invalid were included in analyses of accuracy except where the original user submitted name was not known (the original identification field was added to the database after its launch, and not all original identifications could be retroactively determined), or where the verified species name was “sp” (unknown *Bombus* species); in these latter two cases the records were excluded from analyses.

### Percent agreement analyses

To determine the first measure of accuracy of user identifications, we calculated the percent agreement (represented by Uc/Ut × 100, where Uc is the total number of user submitted records that were verified by experts as being correct and Ut is the total number of user submitted records) for each of the expert reviewed (verified and invalid) species submitted to Bumble Bee Watch, and averaged the individual values for an overall result. This is the same as “recorder success” in Falk et al. (2019), “recorder accuracy” and “recorder identification ability” in Comont & Ashbrook (2017), and “precision” in Roy et al. (2016).

### Veracity analyses

To calculate the second measure of accuracy of user identifications, we calculated the veracity of the user identifications (represented by Uc/E × 100,where Uc is the number of correct user submitted records and E is the total number of expert verified records) for each of the reviewed species submitted to Bumble Bee Watch. This is the same as “recorder accuracy” in Falk et al. (2019), which can be converted to the “miss rate” in Roy et al. (2016) if subtracted from one (i.e., miss rate = 1 − recorder accuracy) (Comont & Ashbrook (2017) do not investigate this metric).

### Comparing percent agreement and veracity

To investigate the overall agreement between the two methods of determining user accuracy (percent agreement and veracity), we used a Fisher’s Exact Test to compare the values calculated for each species. We also subtracted the veracity calculation from the percent agreement to note the difference between the two methods for each species.

### User versus expert identifications

We also tested for significant relationships amongst the user vs expert species identifications overall and for each species individually using Chi-Square tests. For the main analyses (all species being considered at once), we calculated the total number of records per species for every combination of user submitted and expert verified identification (creating a grid with expert species names across the top row and user species names down the first column), and then compared this observed data to the expected data (equation: row total × column total/overall total) to see if the user and expert identifications were related.

For the individual species comparisons, we created new “user_species_x” and “expert_species_x” columns in our data (where “species_x” was replaced with each individual species name; all species names that had been submitted or verified were included) and populated them with a 1 or a 0 depending if that record’s user submitted name or expert identification matched the “species_x” name (=1) or not (=0). These data could then be summarized in a 2 × 2 table, to allow for calculations of observed versus expected for each species to test for relatedness.

When the Chi-square test assumption of all expected values being >0 and 80% of records being >5 were not met, Fisher’s Exact Tests were conducted to provide the significance (*p*) values, except for when all species were being compared at one time, in which case the Monte Carlo method was used to obtain an approximation of the Fisher’s Exact test.

As users might correctly identify a bumble bee simply by chance, we also calculated the Cohen’s Kappa (K) statistic (Landis & Koch, 1977; Lombard, Snyder-Duch & Bracken, 2002; McHugh, 2012). Cohen’s Kappa statistic can take into account chance agreement due to uncertainty by the raters (e.g., in our case, agreement between users and experts even when the users are guessing) (Cohen, 1960; McHugh, 2012). It involves the calculation of probability of observed agreement (i.e., true accuracy) and probability of chance agreement. Kappa values can range from <0 (no observer agreement) to 1 (perfect observer agreement) (Landis & Koch, 1977), although McHugh (2012) suggests that low negative values (at or close to the maximum lowest value of −1) are not meaningful and suggest randomness or errors. While there is no specific agreement on what each Kappa value represents, Lombard, Snyder-Duch & Bracken (2002) and McHugh (2012) suggest that a Kappa of 0.8 or more represents a strong or true agreement while low Kappas (e.g., under 0.60) represent a weak agreement.

We used SPSS version 24 (IBM Corp., Armonk, NY, USA) to conduct all statistical analyses.

### Identification and reporting of at-risk species

As community scientists have previously over-reported rare or at-risk species of other taxa (Dickinson, Zuckerberg & Bonter, 2010; Gardiner et al., 2012; Comont & Ashbrook, 2017), which would lead to a low accuracy rate, as well as making rare species look artificially common, we assessed this for all bumble bee species, as divided into categories based on their IUCN Red List category (IUCN Red List of Threatened Species (IUCN), 2019).

We hypothesized that over-reporting of species would result in a low percent accuracy (i.e., users wanted to find rare species and reported observations as such but were incorrect), and thus we expected a relationship between accuracy and IUCN rank. We used a Generalized Linear Model, with a negative binomial distribution model and log link, followed by pair-wise mean contrast Bonferroni-corrected post-hoc tests, to investigate the relationship between the percent agreement and veracity values for each species (dependent variables, tested separately) and its IUCN rank (predictor variable) (species that are considered Data Deficient were excluded). Percent agreement and veracity values were rounded to the nearest integer for this test.

### Common misidentifications

As some bumble bee species are commonly mistaken for other species, we contrasted the names and numbers of the actual expert verified species for every user submitted species name. We also contrasted the reverse: the number of user submitted species names for each expert verified species name. This was done by plotting user submitted names against the later expert verified names in Microsoft Excel (Microsoft Office 365 ProPlus, Version 1909) to visually compare the number of correct and incorrect identifications.

### User bias

To evaluate the relative contribution of users, and their potential impact or bias on results (e.g., one user contributing a large majority of the records for a specific species, which, if they had better or worse identification accuracy than most users, could affect the results), we calculated the total, maximum, minimum, and average number of users per species, and their overall relative contribution of records by each user to each user submitted name and expert confirmed species. This was conducted in Microsoft Excel.

### Perceived ease of identification

To investigate if accuracy is related to perceived ease of identification, we included a question about the ease of identification in a larger online survey of users and experts about the value of the Bumble Bee Watch program (as discussed in MacPhail, Gibson & Colla (2020)). We recruited participants to complete the anonymous voluntary user survey (Article S1) through information provided in the January 2018 Bumble Bee Watch e-newsletter and posted on social media. The user survey was open for 33 days. The expert survey (Article S2) participants were determined by the authors: the initial list of expert participants was based on verifiers on Bumble Bee Watch who had extensive experience in the *Bombus* field. Invitations were sent to 15 experts in early 2018. Upon analysis of the initial data, to increase the sample size of expert responses, we approached another 17 experts (predominantly non-verifiers on Bumble Bee Watch but who have similar levels of experience in the *Bombus* field) in late 2019. Respondents in this second round of expert surveys had an identical questionnaire to the one sent out in 2018 except for the removal of an invitation and link to complete the general user survey that had been in the initial survey (Article S2).

As part of the survey, we asked respondents a question about species identification difficulty (questions 19 and 20 in Article S1 (users) and questions 14 and 15 in Article S2 (experts)). Species for the respondents’ given geographic area were to be ranked from 1 to 5, where 1 was considered easy to identify and 5 was considered difficult to identify from a photograph (we did not define the quality of the photograph in the survey). Respondents could also choose to select n/a or skip the response for one or more species. If a respondent filled out the ease of identification ranking section for both regions only the data for their identified region was kept.

There were 24 species included in the Eastern region (east of the Mississippi in the United States, and east of Ontario (including Ontario) in Canada) and 34 in the Western region (west of the Mississippi in the United States and west of Manitoba in Canada). We selected these species based on previous Bumble Bee Watch submissions and species listed in Williams et al. (2014), including species with overlapping color patterns, with co-occurring species, common species, and species of conservation interest. Although species were presented for ranking based on geographic area, analyses were conducted independent of region, with data being pooled for each of the 18 species found in both regions.

We collected the data from both surveys using Google Forms, and later exported it to Microsoft Excel. We calculated descriptive statistics of rank responses (including *n*, mean, se mean, median) for each species in Excel. Median ranks were used in analyses as they accounted for unequal numbers of responses due to skipped species and potential outlier ranks. Mann–Whitney *U*-tests were conducted in SPSS to compare the median ease of identification rank responses of users to those of experts for each species (i.e., to see if users and experts agree on difficulty level).

We converted the observed accuracy measures (percent agreement and veracity) to a rank following the assumption that species considered by experts to be easy to identify should have high identification accuracy rates, and those that are difficult to identify should have low accuracy rates. Thus, an accuracy (percent agreement and veracity) of 0–20% was given a rank of five, 21–40% as four, 41–60% as three, 61–80% as two and 81–100% as one.

We then compared these two converted accuracy ranks to the median perceived ease of identification ranks for users and experts. If the percent agreement or veracity calculated from the Bumble Bee Watch dataset, as converted to a rank, matched the median rank value as determined from the survey responses, it was considered a match between perceived ease of identification and actual identification accuracy. We then tested for significant differences between each of the user and expert median ranks as compared to the percent agreement and accuracy rank (to see if perceptions of ease of identification were the same as observed accuracy), for all species combined, using Chi-Square tests or, if assumptions of expected values being >0 or 80% of values >5 were not met, Fisher’s Exact Tests.

This survey of users and experts was approved by York University’s Faculty of Environmental Studies (November 2017, no reference number (internal approval by Drs. Colla and Meyers)) and the data re-approved for use by York University’s Office of Research Ethics (September 2019, ref# STU 2019-097).

## Results

### Data collection

We obtained 22,159 reviewed bumble bee records (i.e., verified and invalid submissions to Bumble Bee Watch with a known original user submitted name, excluding those verified as “sp” by experts) for our analyses. After expert review, this represented 39 species and three other species categories (two species complexes and an unknown/not a bumble bee category) (hereafter all referred to as species) being represented in the records (Table 2).

**Table 2:** The number and relative abundance of the bumble bee species records reviewed by experts and the number of species originally identified by users for each expert-reviewed species and vise versa. Note that *Bombus bohemicus* was not correctly identified by any users so the count of one original species identification per verified species is not including an accurate submission. Records verified as “sp” (unknown *Bombus*) were excluded from our data set.

*Bombus* species	Total # reviewed records	Relative abundance of reviewed records (%)	# Original species names submitted by users per reviewed species	# Verified species names for each user-submitted species name
*affinis*	345	1.56	3	12
*appositus*	198	0.89	13	16
*auricomus*	105	0.47	10	16
*bifarius*	1,387	6.26	19	22
*bimaculatus*	1,340	6.05	21	9
*bohemicus*	2	0.01	1	5
*borealis*	328	1.48	14	9
*caliginosus*	60	0.27	5	9
*centralis*	476	2.15	14	9
*citrinus*	94	0.42	16	10
*crotchii*	9	0.04	2	10
*cryptarum*	107	0.48	7	1
*fervidus*	563	2.54	27	32
*flavidus*	125	0.56	14	8
*flavifrons*	919	4.15	26	18
*franklini*	0	0.00	0	2
*fraternus*	20	0.09	1	9
*frigidus*	20	0.09	6	8
*griseocollis*	1,520	6.86	22	17
*huntii*	659	2.97	15	11
*impatiens*	4,205	18.98	28	18
*insularis*	240	1.08	16	19
*kirbiellus*	3	0.01	1	23
*melanopygus*	599	2.70	18	23
*mixtus*	731	3.30	21	13
*morrisoni*	46	0.21	6	10
*nevadensis*	442	1.99	13	20
*occidentalis*	242	1.09	7	19
*pensylvanicus*	93	0.42	10	13
*perplexus*	297	1.34	16	10
*rufocinctus*	937	4.23	32	28
*sandersoni*	21	0.09	7	13
*sitkensis*	113	0.51	11	6
sp.	n/a	n/a	n/a	37
*suckleyi*	1	0.00	1	14
*sylvicola*	46	0.21	7	17
*ternarius*	1,154	5.21	17	11
*terricola*	279	1.26	12	20
Unknown (non-*Bombus*)	2,037	9.19	41	n/a
*vagans*	539	2.43	21	14
*vagans, sandersoni* or *perplexus*	292	1.32	18	n/a
*vandykei*	69	0.31	12	7
*variabilis*	0	0.00	0	6
*vosnesenskii*	1,112	5.02	15	9
*vosnesenskii* or *caliginosus*	384	1.73	18	1
Grand Total	22,159	100.00	42	43

Experts verified that there were 11,690 records (52.76%) that were correctly identified to species upon initial submission by users. An additional 2,037 records (9.19%) were invalid (not a bumble bee), while the remaining 8,432 records (38.05%) were bumble bees that had been incorrectly identified by the user, for a total of 10,469 (47.24%) incorrect user identifications overall.

The most commonly found species after expert review were *B. impatiens* Cresson, 1863 (4,205 records, 18.98% of all reviewed submissions), followed by unknown/not a bumble bee (2,037 records, 9.19%) and *B. griseocollis* (DeGeer, 1773) (1,520 records, 6.86%) (Table 2). The least commonly found species were *B. suckleyi* Greene, 1860 (1 record, 0%) *B. bohemicus* Seidl, 1838 (2 records, 0.01%), and *B. kirbiellus* Curtis, 1835 (3 records, 0.01%) (Table 2).

Records were submitted from all 13 Canadian provinces and territories and 49 US states (including District of Columbia) where bumble bees are found (Hawaii does not have any bumble bees). The numbers submitted per jurisdiction were variable, ranging from 6,147 records in Ontario (27.74% of all records) to two records from each of West Virginia and Yukon (0.01% of all records each) (Table S1). The top four jurisdictions accounted for 65.03% of all records (Table S1).

A total of 342 individuals responded to the Bumble Bee Watch user survey, representing 5.4% of all Bumble Bee Watch participants (individuals who had submitted records to the program as of the survey close date) (MacPhail, Gibson & Colla, 2020). Fifteen of the 32 experts (46.9%) invited responded to the expert survey, with 8 selecting the Eastern Region as the area they were most familiar with and 7 selecting the Western Region. Nine of the 15 responding experts (60%) had previously verified records on Bumble Bee Watch (MacPhail, Gibson & Colla, 2020). As respondents could skip questions, the total number of respondents (both users and experts) providing their ranked perception of ease of identification varied from two to 13 per species.

### Percent agreement analyses

There was an average of 53.20% percent agreement (number of records correctly identified by users compared to all user submitted records) (i.e., Uc/Ut × 100) across all species, with a range of 0 (*B. bohemicus, B. franklini* (Frison, 1921), and *B. kirbiellus*, with no correct user submissions) to 100% (*B. cryptarum* (Fabricius, 1775), with all user submissions having the correct identification) per species (Table 3).

**Table 3:** A comparison of the number of records submitted by users, number that were correctly identified, number verified by experts, the percent agreement and veracity rank and related statistical values. The percent agreement and veracity ranks were calculated as follows: 1, 81–100%; 2, 61–80%; 3, 41–60%; 4, 21–40%; 5, 0–20%. Species or species equivalents (e.g., non*-Bombus* observations) that were not available for selection by users were not included in analyses of percent agreement or veracity. *Bombus variabilis* and *B. franklini* were removed from analyses involving veracity as there were no verified records in the dataset. Observations submitted by users as unidentified *Bombus* sp. were removed from the original dataset but experts could later identify other submissions as *Bombus* sp.

*Bombus* species	# Correct user-submissions (Uc)	# Total user-submissions (Ut)	Percent agreement (Uc/Ut × 100)	Percent agreement rank	# Total expert-verified records (E)	Percent veracity (Uc/E × 100)	Veracity rank	Difference b/w percent agreement, veracity
*affinis*	333	762	43.70	3	345	96.52	1	52.82
*appositus*	138	176	78.41	2	198	69.70	2	−8.71
*auricomus*	72	189	38.10	4	105	68.57	2	30.48
*bifarius*	985	1,228	80.21	2	1,387	71.02	2	−9.20
*bimaculatus*	800	1,166	68.61	2	1,340	59.70	3	−8.91
*bohemicus*	0	27	0.00	5	2	0.00	5	0.00
*borealis*	191	220	86.82	1	328	58.23	3	−28.59
*caliginosus*	43	232	18.53	5	60	71.67	2	53.13
*centralis*	291	381	76.38	2	476	61.13	2	−15.24
*citrinus*	43	100	43.00	3	94	45.74	3	2.74
*crotchii*	8	19	42.11	3	9	88.89	1	46.78
*cryptarum*	40	40	100.00	1	107	37.38	4	−62.62
*fervidus*	368	643	57.23	3	563	65.36	2	8.13
*flavidus*	57	87	65.52	2	125	45.60	3	−19.92
*flavifrons*	461	656	70.27	2	919	50.16	3	−20.11
*franklini*	0	5	0.00	5	0	n/a	n/a	n/a
*fraternus*	20	44	45.45	3	20	100.00	1	54.55
*frigidus*	14	55	25.45	4	20	70.00	2	44.55
*griseocollis*	921	1,283	71.78	2	1,520	60.59	2	−11.19
*huntii*	446	531	83.99	1	659	67.68	2	−16.31
*impatiens*	2,539	2,994	84.80	1	4,205	60.38	3	−24.42
*insularis*	75	216	34.72	4	240	31.25	4	−3.47
*kirbiellus*	0	134	0.00	5	3	0.00	5	0.00
*melanopygus*	309	517	59.77	3	599	51.59	3	−8.18
*mixtus*	437	491	89.00	1	731	59.78	3	−29.22
*morrisoni*	32	60	53.33	3	46	69.57	2	16.23
*nevadensis*	333	457	72.87	2	442	75.34	2	2.47
*occidentalis*	218	372	58.60	3	242	90.08	1	31.48
*pensylvanicus*	45	124	36.29	4	93	48.39	3	12.10
*perplexus*	98	118	83.05	1	297	33.00	4	−50.05
*rufocinctus*	422	906	46.58	3	937	45.04	3	−1.54
*sandersoni*	4	127	3.15	5	21	19.05	5	15.90
*sitkensis*	38	67	56.72	3	113	33.63	4	−23.09
sp.	n/a	4,876	n/a	n/a	n/a	n/a	n/a	n/a
*suckleyi*	1	53	1.89	5	1	100.00	1	98.11
*sylvicola*	24	170	14.12	5	46	52.17	3	38.06
*ternarius*	727	791	91.91	1	1,154	63.00	2	−28.91
*terricola*	198	268	73.88	2	279	70.97	2	−2.91
Unknown (Non-*Bombus*)	n/a	n/a	n/a	n/a	2,037	n/a	n/a	n/a
*vagans*	239	516	46.32	3	539	44.34	3	−1.98
*vagans, sandersoni* or *perplexus*	n/a	n/a	n/a	n/a	292	n/a	n/a	n/a
*vandykei*	25	48	52.08	3	69	36.23	4	−15.85
*variabilis*	0	141	0.00	5	0	n/a	n/a	n/a
*vosnesenskii*	694	868	79.95	2	1,112	62.41	2	−17.54
*vosnesenskii* or *caliginosus*	1	1	100.00	1	384	0.26	5	−99.74
Total	11,690	22,159	52.76	3	22,159	52.76	3	0.00
Average	278.33	515.33	53.20	2.86	503.61	55.86	2.73	0.00

**Notes:**

Ut, total number of records per species submitted by users to the Bumble Bee Watch citizen science program.

Uc, number of records that were correctly identified by users according to experts.

E, total number of verified records per species overall.

Percent agreement: Uc/Ut × 100.

Veracity: Uc/E × 100.

### Veracity analyses

The average veracity rate (number of records correctly identified by users compared to all verified records) (Uc/E × 100) was 55.86%, although this varied by species, ranging from 0 (*B. bohemicus* and *B. kirbiellus*, with no records submitted by users with the correct identification despite two and three records verified as being present in the data per species, respectively) to 100% (*B. fraternus* (Smith, 1854) and *B. suckleyi*, with all verified records having been submitted with the correct identification by the users) (Table 3).

### Comparing percent agreement and veracity

There was no significant difference in percent agreement as compared to veracity when looking at all species together (Fisher’s Exact Test = 19.829, df = 16, *p* = 0.075). However, there were differences noted between the two metrics for all species except for *B. bohemicus* and *B. kirbiellus*. The results of subtracting veracity from percent agreement ranged from −62.2 (*B. cryptarum*) to 98.11% (*B. suckleyi*) (Table 3).

### User versus expert individual species identifications

The difference in user submitted names versus expert verified names was significant over all species (i.e., the user and expert responses were not related) (Fisher’s Exact Test = 65,217.914, df = 1,681, *p* < 0.001, κ = 0.5). The user versus expert identification agreement, when investigated for each species separately, was also significantly different for all species except for *B. bohemicus* (which had no correct original identifications by users although there were misidentified submissions that were later verified to be *B. bohemicus*) (Table S2).

For some species, the percent agreement or veracity measures between user and expert identifications is likely not a true agreement and due in large part to chance: for example, the Kappa values of 0.21–0.40 suggested *B. caliginosus* (Frison, 1927), *B. frigidus* Smith, 1854, *B. insularis* (Smith, 1861), and *B. sylvicola* Kirby, 1837 were all minimal or fair agreement (Table S2). For other species, the percent agreement is more likely a true agreement between users and experts and not just agreement due to chance: for example, 14 species all had moderate or substantial agreement (Kappa values from 0.61 to 0.80). Two species, *B. bohemicus* and *B. kirbiellus*, had 0% agreement, which is supported by Kappa values of zero (Table S2).

### Identification and reporting of at-risk species

The level of rarity, according to the IUCN Red List ranking, has a significant predictive effect on percent agreement (Wald Chi-Square = 19.821, df = 3, sig < 0.0002 ). The only significant differences were between IUCN rank 1 (Least Concern) and rank 3 (Endangered) (*p* = 0.002), and rank 1 and rank 4 (Critically Endangered) (*p* = 0.002), with percent agreement values decreasing as the IUCN rank increases (Tables S3 and S4). The level of rarity also has a significant predictive effect on veracity (Wald Chi-Square = 141.320, df = 3, sig < 0.0001), but the opposite effect as percent agreement. All pair-wise comparisons were significantly different (*p* < 0.05) except for IUCN ranks 3 and 4 (*p* = 1.00), with higher values of veracity as the IUCN rank increases (Tables S3 and S4). Table S4 presents the means and estimates of the marginal means for each IUCN rank for both percent agreement and veracity.

### Common misidentifications

The number of expert verified species names that an original user submitted name turned out to be ranged from 32 to 1, depending on the species (Table 2; Table S5). Records submitted by users as *B. fervidus* (Fabricius, 1798) resulted in 32 different verified species while *B. rufocinctus* Cresson, 1863 resulted in 28 different verified species. At the opposite end of the spectrum, *B. bohemicus* user submitted records led to 5 different verified species, *B. franklini* to 2 different verified species, and only 1 for *B. cryptarum*.

The number of user submitted species per verified species ranged from 32 to 0 (Table 2; Table S5). The highest was *B. rufocinctus* (32 species as identified and submitted by users) followed by *B. impatiens* (28 species submitted) and *B. fervidus* (27 species submitted). The lowest was *B. crotchii* Cresson, 1878 (2 species submitted) followed by *B. bohemicus*, *B. fraternus*, *B. kirbielllus* Curtis, 1835 and *B. suckleyi* (1 species submitted for each); no records were verified as *B. franklini* or *B. variabilis* Cresson, 1872 despite user submissions as such. Additionally, 41 species had been identified and submitted by users for records that were not actually of bumble bees (i.e., invalid records once reviewed by experts). Note that the above summaries (Table 2) deal with the actual number of incorrect species names; it is not the same as the accuracy of identification, which can be determined by evaluating the relative percent agreement and veracity values in Table 3.

### User bias

The 22,159 records used in these analyses were submitted by 4,912 different users. The number of records submitted per user ranged from 1 record to 448, with a mean of 4.5 ± 0.27 records per person. The number of unique users per reviewed species varied, but an average of 162.9 ± 34.89 people submitted records for each reviewed species (Table S6). This ranged from 1 user (*B. bohemicus* and *B. suckleyi*) to 1,447 users (*B. impatiens*). The average number of records per reviewed species submitted per user ranged from 1 (no standard error, single observation only) (*B. kirbiellus* and *B. suckleyi*) to 6.51 ± 0.89 records (*B. bifarius* Cresson, 1878). The maximum number of observations per species per user ranged from 1 (33.3% of all records of that species for *B. kirbiellus* and 100% all records of that species for *B. suckleyi*) to 271 (6.73% of all records for *B. impatiens*).

The maximum relative numbers of records submitted by one user per verified species (i.e., the maximum contribution per person per verified species) (Table S6) ranged from 0.98% (unknown, not a bumble bee) and 2.86% (*B. vosnesenskii* or *caliginosus* group) through to 100% (*B. bohemicus* and *B. suckleyi*), with the overall contribution of 2.02% per user per species. However, sample size effects these results; for example, there was only one user who submitted verified records each of *B. bohemicus* and *B. suckleyi*. When at least ten users had submitted verified records of a species, the range went from 0.98% (unknown, not a bumble bee) to a maximum contribution per user per species of 40.0% for *B. frigidus* (Table S6).

### Perceived ease of identification

When we compared the percent agreement and veracity ranks (Table 3) per species to the median perception of ease of identification ranks (Table 4), we found that neither accuracy rank was statistically different as compared to the user median perception rank (Fisher’s Exact Test = 7.562, df =8, *p* = 0.396 and Fishers Exact Test = 5.016, df = 8, *p* = 0.836, respectively). The percent agreement was significantly different from the expert median perception rank (Fisher’s Exact Test = 27.074, df = 16, *p* = 0.002) but the veracity was not (Fisher’s Exact Test = 16.694, df = 16, *p* = 0.239, respectively).

**Table 4:** Comparison of the rankings of perception of ease of identification between users and experts in the Bumble Bee Watch survey. Species that occurred in both the Eastern and Western regions of the surveys had their responses combined. A median response of 1 indicated respondents perceived the species to be easy to identify, with the range extending to 5, which was for species perceived to be difficult to identify. M–W *U* stat, Mann–Whitney *U*-test Statistic; *p*-value, Exact Significance (2-tailed); Sig?, significant difference between users and experts?; n, number of respondents; perceived harder?, who (user or expert) perceived the species identification to be hardest? User and Expert ranks (percent agreement ranks) are as follows: 1, Easy (81–100% accuracy); 2, Somewhat Easy (61–80% accuracy); 3, Medium (41–60% accuracy); 4, Somewhat Difficult (21–40% accuracy); 5, Difficult (0–20%). *Bombus huntii* and *B. sitkensis* were not included in the user or expert survey, and no verified records of *B. jonellus* were found in the database.

	*affinis*	*appositus*	*auricomus*	*bifarius*	*bimaculatus*	*bohemicus*	*borealis*	*caliginosis*	*centralis*	*citrinus*	*crotchii*	*cryptarum*	*fervidus*	*flavidus*
M–W *U* stat	176.5	42	905	58.5	160.5	633.5	279	99	76	393	30.5	35	403.5	281.5
*p*-value	0.003	0.001	0.437	0.005	0.001	0.058	0	0.139	0.02	0.001	0	0.054	0	0.899
Sig?	yes	yes	no	yes	yes	no	yes	no	yes	yes	yes	no	yes	no
perceived harder?	user	user	n/a	user	user	n/a	user	n/a	user	user	user	n/a	user	n/a
expert *n*	7	6	12	6	7	10	12	6	6	11	6	4	13	6
expert median	1	1	3	2	1	4	1	4	2	3	1	2	1	5
expert mean	1.6	1.5	3.3	2.0	1.3	3.4	1.8	4.3	2.2	2.8	1.8	2.3	1.6	4.0
expert se mean	0.4	0.5	0.3	0.4	0.3	0.5	0.4	0.2	0.5	0.4	0.5	0.8	0.3	0.7
user *n*	137	54	174	57	142	140	162	53	55	154	40	42	190	96
user median	3	3	4	4	3	4	4	4	4	4	4	4	3	5
user mean	3.2	3.3	3.5	3.5	3.1	4.1	3.8	3.7	3.4	4.0	3.8	3.7	3.4	4.4
user se mean	0.1	0.1	0.1	0.1	0.1	0.1	0.1	0.1	0.1	0.1	0.1	0.1	0.1	0.1

	*flavifrons*	*franklini*	*fraternus*	*frigidus*	*griseocollis*	*impatiens*	*insularis*	*jonellus*	*kirbiellus*	*melanopygus*	*mixtus*	*morrisoni*
M–W *U* stat	116	23	64	438	289	572.5	665.5	34	48	63	68.5	93
*p*-value	0.079	0.006	0	0.032	0	0	0.127	0.063	0.456	0.007	0.073	0
Sig?	no	yes	yes	yes	yes	yes	no	no	no	yes	no	yes
perceived harder?	n/a	user	user	user	user	user	n/a	n/a	n/a	user	n/a	user
expert *n*	6	4	9	10	12	13	12	4	2	6	5	8
expert median	2	2	1	3	1	1	4	2	5	2	1	1
expert mean	2.3	2.0	1.4	3.4	1.2	1.3	3.4	2.3	5.0	2.0	2.2	1.8
expert se mean	0.6	0.6	0.3	0.4	0.2	0.2	0.5	0.8	0.0	0.4	0.7	0.4
user *n*	66	46	134	142	200	209	147	39	84	56	54	132
user median	4	4	4	4	4	3	4	4	5	4	4	4
user mean	3.4	3.6	4.0	4.1	3.2	2.7	4.2	3.6	4.3	3.4	3.6	4.0
user se mean	0.1	0.1	0.1	0.1	0.1	0.1	0.1	0.2	0.1	0.1	0.1	0.1

	*nevadensis*	*occidentallis*	*pensylvanicus*	*perplexus*	*rufocinctus*	*sandersoni*	*suckleyi*	*sylvicola*	*ternarius*	*terricola*	*vagans*	*variabilis*	*vandykei*	*vosnesenski*
M–W *U* stat	275.5	68	874.5	447.5	1092.5	468	84	52.5	349	38.5	891.5	153	96	74
*p*-value	0.001	0.011	0.306	0.004	0.612	0.013	0.279	0.041	0	0	0.59	0.465	0.118	0.022
Sig?	yes	yes	no	yes	no	yes	no	yes	yes	yes	no	no	no	yes
perceived harder?	user	user	n/a	user	n/a	expert	n/a	user	user	user	n/a	n/a	n/a	user
expert *n*	10	6	12	11	13	11	5	5	12	7	11	4	6	6
expert median	2	1	3	3	4	5	4	3	1	1	3	4	3	1
expert mean	2.2	1.7	3.1	2.6	3.4	4.7	3.6	3.0	1.3	1.0	3.3	3.5	2.7	1.7
expert se mean	0.4	0.4	0.4	0.5	0.4	0.2	0.2	0.0	0.2	0.0	0.5	1.0	0.6	0.4
user *n*	138	59	176	159	183	146	48	46	179	120	179	97	50	55
user median	4	3	4	4	4	4	4	4	3	4	4	5	4	3
user mean	3.8	3.1	3.5	4.1	3.7	4.1	4.0	3.7	3.2	3.6	3.6	4.3	3.5	3.0
user se mean	0.1	0.2	0.1	0.1	0.1	0.1	0.1	0.1	0.1	0.1	0.1	0.1	0.1	0.2

There was a significant difference between users’ and experts’ perceived ease of identification (Table 4) for 24/40 species (60%). Users usually considered it harder to identify species than experts, as 23/24 (95.3%) of the species with significant differences had a higher median score given (i.e., more difficult to identify) by users than experts (Table 4).

Three of the 40 species (*B. flavidus* Eversmann, 1852, *B. kirbiellus, B. variabilis*) were considered hardest by users to identify, having a median rank of 5—Difficult, while no species were considered easy or somewhat easy (i.e., none had a median rank of 1 or 2) to identify by users, 8 species had a median rank of 3—medium (Table 4). Three of the 40 species (*B. flavidus, B. kirbiellus, B. sandersoni* Franklin, 1913) were considered hardest by experts to identify having a median rank of 5—Difficult, while 15/40 were given a median rank of 1—Easy (Table 4).

## Discussion

The accuracy of initial identification is important for determining the utility and quality of community science-collected data (Gardiner et al., 2012). We found that records submitted by Bumble Bee Watch community scientists have an overall percent agreement of 52.76% (range 0–100%, average 53.20%), when comparing the number of correct user submitted records (as determined by expert review) to the total number of user submitted records (i.e., Uc/Ut × 100). This compares closely to the overall “recorder success” for BeeWatch at 59% (range 4–84%, average 60%) and Blooms for Bees at 40% (range 0–100%, average 21%) (Falk et al., 2019), “recorder accuracy” and “recorder identification ability” for BeeWatch at 59% (range 51–64%) (Comont & Ashbrook, 2017), as well as “precision” at 44% (range 0–86%) for Big Bumblebee Discovery (Roy et al., 2016).

Our overall veracity (proportion of all verified records correctly identified by users) (i.e., Uc/E × 100) was 52.76%, (range 0–100%, average 55.86%). This compares to an overall value of 49% (range of 42–78%, average 45%) in Bee Watch and 44% (range of 0–100%, average 39%) in Blooms for Bees (Falk et al., 2019) and 63% (range 0–83%, average 45%) in Big Bumblebee Discovery (Roy et al., 2016) (note that Comont & Ashbrook (2017) did not investigate this metric), suggesting that Bumble Bee Watch participants miss fewer identifications than in other programs.

While we used two different ways of calculating accuracy, percent agreement and veracity, we found that there was no significant difference between them over all species combined, although there were large differences observed for some species (e.g., observers captured 97% of all verified *B. affinis* Cresson, 1863 records in their submissions, although their percent agreement of identifications was low with only 44% of their total *B. affinis* submissions being correct).

While the interpretation of Cohen’s Kappa statistic is not consistent across researchers, our Kappa values do suggest that the percent agreement and veracity values calculated for a number of species in our study should be interpreted with caution as the agreement between users and experts is likely a result of chance. For instance, of the 41 species we calculated the statistic for in our study, 9 had Kappa values of less than 0.40, which is considered only a slight or fair agreement by Landis & Koch (1977) and no or minimal agreement by McHugh (2012), and another 17 species had values less than 0.60, which while considered a moderate agreement by Landis & Koch (1977), is considered a weak agreement by McHugh (2012). These low values of agreement (high involvement of chance) may be due to the small sample size observed with some species, and/or the small number of correct observations submitted by users.

While ten of the 42 species in our analyses (*B*. *bifarius, B. borealis* Kirby, 1837, *B. cryptarum*, *B. huntii*, *B. impatiens*, *B. mixtus* Cresson, 1878, *B. perplexus* Cresson, 1863, *B. ternarius* Say, 1837, *B. vosnesenskii* Radoszkowski, 1862, and *B. vosnesenskii or caliginosus*) were at or above the suggested level of 80% percent agreement that would eliminate or reduce the need for expert review (Aceves-Bueno et al., 2017), the remaining 32 species were below this level. Falk et al. (2019) had two (of 22) species above the 80% threshold in the BeeWatch program and one (of 24) species in the Blooms for Bees programs, while Roy et al. (2016) had two (out of 5 color groups) above the 80% threshold for the Big Bumblebee Discovery program. It is not uncommon for community science programs to be under the 80% threshold, as Aceves-Bueno et al. (2017) found that only 52% of programs were at or above that value. This does, however, reinforce the need for expert-review in our program and others that involve bumble bee identification by community scientists.

Critically endangered and endangered species were more likely to be misidentified in our study than species considered to be at least risk of extinction when percent agreement was evaluated, but the opposite trend was seen with veracity, where, except for between endangered and critically endangered species, levels of veracity increased as risk of extinction increased, although there was a low sample size in the higher risk of extinction levels. The low percent agreement could be because some at-risk species are more cryptic or difficult to identify as compared to other at-risk species and common species (i.e., uncommonness may or may not result in difficulty in identification). There is also increasingly more guidance and education about how to identify some rare species, and many of these species (e.g., *B. affinis, B. occidentalis* Greene, 1858) are targeted in searches and programs for their conservation; while these targeted searches may increase the total veracity by capturing more of the actual occurrences in the initial identifications, it may also result in users misidentifying more observations due to a desire to find the rare species or because they recognize that species name in the list of options, therefore dropping the percent agreement (number of correct observations compared to all submitted observations of that species).

We anticipated that experts in bumble bee biology, particularly those with extensive identification experience, would be better able to rank the difficulty of identifying species by photos than users, which we did not clearly see in this study. We also anticipated that species that are perceived as easy to identify would have a higher identification accuracy. Although there was a significantly different relationship with expert rank of ease of identification and actual observed percent agreement of identification rates in this article (i.e., the expert prediction did not match the observed percent agreement), the veracity was not different from the expert perception (i.e., the expert view was similar to the veracity observed over all records). This difference in findings could be related to users misidentifying a number of species in their submitted identifications and thus skewing the percent agreement results as compared to the overall perception of experts. There was no difference with users and observed percent agreement or veracity, although the predicted range rarely met the observed range of percent agreement. It is also possible that experts do not fully understand how difficult users find the identification process and the perceptions of the two groups could be quite different and the findings only relevant to that specific group.

Experts generally ranked species as easier to identify than users. This may be because experts have a lot more experience and training, look for features that users do not, and know more “exceptions” to the typical or standard appearance, and thus have less confusion or hesitation over identifications than users may have. The respondents to the user survey were not entirely representative of contributors to Bumble Bee Watch overall, with the respondents tending to submit more records than users overall (MacPhail, Gibson & Colla, 2020). It is possible that the rankings would be different than that given by a truly representative sample of contributors, particularly those who only submitted one record. However, many users may not actually know enough to be willing to offer a ranking; in our user survey, the number of respondents who ranked a species ranged from 39 to 209 respondents per species out of the 342 respondents in total. Similarly, a low number of experts contributed to ranks of some species in the expert survey, ranging from 2 to 13 respondents per species out of 15 total respondents. It is possible that if a larger sample of both users and experts were obtained, results closer to observed may be obtained. Additionally, no photographs or illustrations were provided for the species in the survey, so users may have had difficulty assigning a rank as they were unsure what that bee looked like; future investigations should provide standardized photos or illustrations (e.g., color pattern guides as used in Williams et al. (2014)). In the future, experts could be specifically asked their perceptions on how easy or hard it is for the average user to identify the species, rather than their own perceptions.

The range in numbers of user submitted names per expert assigned species, and vise versa, was extensive. Mimicry and cryptic coloration patterns may cause greater difficulty in accurate species identification than species with consistent patterns. Identification guides do show the variations present in some species, with *B. rufocinctus* being the most diverse (Williams et al., 2014). Additionally, some species may have had more incorrect species names because they are a wide-ranging species and thus more potential for confusion with other co-occurring species in different parts of its range. These are two areas that could be investigated further.

As noted above, some species can be hard to identify, with specific photo angles needed to showcase identifying characteristics, and/or with characteristics best seen under a microscope using a physical specimen. In some cases, experts may still be able to identify the specific species without a microscope using just the photos provided, often based on gestalt or intuition built on past experience; this may also explain why experts in our study perceived similar-looking species (e.g., *B. sandersoni* and *B. vagans* Smith, 1854) as being at different levels of identification difficulty (levels 5 and 3, respectively).

Bumble Bee Watch uses two species complexes or groups to help in situations when the observation can be narrowed down to one of a few similar-looking species, but confirmation of the exact species cannot be made. These include *B. vagans, B. sandersoni* and *B. perplexus* (the two-striped group) and *B. vosnesenskii* and *B. caliginosus* (the yellow-faced group). When the percent agreement is calculated with the allowance that any of the user-submitted species is correct if it is verified as being either that species or one of the species in the associated complex, the percent agreement increases, sometimes drastically, as compared to strictly considering the single species in isolation.

For example, of the 516 records that had been submitted as *B. vagans*, 333 were verified as either being *B. vagans, B. sandersoni, B. perplexus*, or part of the complex, leading to a potential 64.53% percent agreement if any of those responses were to be considered correct, as compared to original comparison of 239 being verified as *B. vagans* alone (46.32% percent agreement). For *B. sandersoni*, the percent agreement increases to 38.58% from the original 3.15% with the extra allowance, and for *B. perplexus*, 86.44% from the original 83.02%. Similarly, for *B. vosnesenskii*, the percent agreement increases to 96.20% from 79.95% if you include all records verified as *B. vosnesenskii, B. caliginosus*, or the complex, and for *B. caliginosus*, 95.26% from 18.53%. This suggests that, at least for some hard to identify species, users may not be as “wrong” or inaccurate as the main calculation shows (i.e., they were “close” to the correct identification), an additional area for consideration.

The Bumble Bee Watch platform has a system of checks in place that only allow for species known to be in that provincial/territorial/state jurisdiction to be entered, reducing the number of potential incorrect species options for species. While the program’s interactive identification key and filter also allow for greater accuracy, it also introduces a potential source of error related to the identification of male specimens: males do not always follow the same colour patterns as females, but the program’s keys and filters focus solely on the female patterns.

To increase the percent agreement and veracity of community science-collected data, professional scientists may install quality control and assurance methods (Kosmala et al., 2016; Freitag, Meyer & Whiteman, 2016; Aceves-Bueno et al., 2017). Bumble Bee Watch could update its various existing filters and checkpoints, or what have also been referred to as “automated error checking capabilities” (Crall et al., 2011), or “smart filter” systems (Freitag, Meyer & Whiteman, 2016), throughout the identification key for at-risk species, males of species that differ in appearance from the females, and for other species that are frequently misidentified. A similar validation method is used by Project FeederWatch, a community science program where a system of semi-automated, novel filters for each US state or Canadian province and territory dictates the submission of records by users (Bonter & Cooper, 2012). The Project FeederWatch program uses historical data to help detect potential errors in bird identification, for example, records submitted in locations outside of known ranges (Bonter & Cooper, 2012). This system had an initial accuracy of 97.7% by participants over three consecutive years (Bonter & Cooper, 2012). Bumble Bee Watch currently has a data validation pathway during identification to restrict based on range, but could add in prompts with tips and checks to help users become aware of potential mimics and to discern the differences in the frequently misidentified species.

For our analyses, we removed records that could not be determined to species by experts; this was often due to the submission of poor-quality photos or photos missing key features/angles needed for identification. It is possible that these unknown bumble bee species had in fact been correctly identified by the user but the records were not verifiable. To increase the amount of verifiable records, additional fact sheets and tutorials can be provided. When users have a better understanding of the specific features needed for identification of each bumble bee species, they can take photos that allow for easier and more accurate expert identifications (Suzuki-Ohno et al., 2017). Continued participation in the program could also have users increase their accuracy, particularly when they have previously submitted less than ten records (Aceves-Bueno et al., 2017; Falk et al., 2019). Supporting the user experience would increase retention and participation rates, which would improve the dataset.

While observer bias could influence percent agreement, particularly when the number of observers and/or the number of records are small, it is unlikely to be a problem in our study for most species. The vast majority of the 4,912 users only submitted one or two photos, and their overall contribution was also low per species. There are a few exceptions where observer bias would likely play a role, such as for *B. bohemicus* and *B. suckleyi* as only 1 user submitted verified records of each, as well as *B. kirbiellus* with 3 users and *B. crotchii* with 5 users. While *B. frigidus* had 12 users who submitted verified records, the most frequent user had submitted 40% of the records, which could influence the accuracy if (s)he was a high or low skill level for identification.

An assumption behind Cohen’s Kappa is each observer (in our case users and experts) independently classify their observations. From an analysis of our data (not presented in the text), 673 of the 22,159 total reviewed records (3.04%) were self-verified (i.e., submitted by and reviewed by the same individual), which would violate the assumption. However, since it is such a small value, and the verifications do not usually occur at the same time as the original submissions, we do not believe it would cause an impact on the analyses.

A current challenge with the Bumble Bee Watch program, and one other bumble bee community science programs face (Comont & Ashbrook, 2017; Falk et al., 2019), is the delay that may exist in the time it takes a record to be reviewed by an expert and the user receiving confirmation of the identification; this was noted as one of the most common drawbacks to the program in a recent user survey (MacPhail, Gibson & Colla, 2020). While each record currently is reviewed by experts regardless as to its probable accuracy, the current structure of the verification side of the database allows for accurate identifications to be reviewed and generalized feedback (often just a brief thank you) to be given much faster than when the identification needs to be changed or personalized feedback given (although currently even the generalized feedback needs to be expert generated versus automatically generated as is the case with BeeWatch (Falk et al., 2019)). Increasing the initial user accuracy on Bumble Bee Watch therefore means record validation may proceed more quickly and efficiently, as experts would spend less time correcting an identification and providing feedback to participants about why the identification is wrong, and more time processing submissions, thus providing a faster turnaround time from the time a participant submits a record to the time they get a confirmation. If the percent agreement for a species is reliably high, over time it may be possible for experts to skip the review process for those records and/or verify a sub-sample only, thus further freeing up resources to focus on the more commonly misidentified species.

An assumption of Bumble Bee Watch is that the expert reviewers are always “correct”. However, experts are not always correct or consistent (Stribling et al., 2008; Kosmala et al., 2016; Austen et al., 2016, 2018; Suzuki-Ohno et al., 2017). Misidentifications may have little impact but could have serious repercussions, particularly related to noting species declines, conserving endangered species, tracking invasive or harmful species, and other related environmental protections and conservation activities (Austen et al., 2016; Stribling et al., 2008). It is therefore important to make sure taxonomic identifications are validated (Packer et al., 2018). More investigations into the accuracy and consistency of expert identifications of bumble bees, particularly from field photos, should be conducted, and additional quality control procedures implemented in the Bumble Bee Watch database.

The Bumble Bee Watch program has thus far recorded 39 unique species from across North America. There are 46 species listed as being found in North America by Williams et al. (2014): the missing ones include *B. distinguendus* Morawitz, 1869, *B. franklini*, *B. hyperboreus* Schönherr, 1809, *B. jonellus, B. neoboreus* Sladen, 1919, *B. polaris* Curtis, 1835, and *B. variabilis*. It is not surprising that these species are missing. *Bombus franklini* is suspected to be extinct, previously occupying an extremely narrow range in southern Oregon to northern California (Williams et al., 2014). *Bombus variabilis* is one of the rarest bumble bees in North America and has been extremely infrequently seen (Williams et al., 2014). All the other missing species are only found in the tundra/taiga in the far north (Williams et al., 2014), where there are few humans let alone participants with the Bumble Bee Watch program. That said, there may be records of these species still awaiting review on Bumble Bee Watch, or that have previously been verified as an unknown bumble bee species, as these species are frequently difficult to identify, particularly from photos. We have already encountered a few photos from the far north that we cannot comfortably identify: in some cases a tentative identification was discussed in the comments (e.g., bee record 3,547 is a tentative *B. hyperboreus* and bee record 4,922 is a tentative *B. polaris*) while others were generally identified simply as “sp” (e.g., bee records 15,468, 15,540). The encouragement and allowance of more photos or videos to be submitted per record (the current maximum is 3 photos), and the additional guidance of best features to photograph per species, may allow further identifications to occur in the future. Users submitting images of these potential species could also be contacted in order to obtain more photos and/or physical specimens for confident identifications to be made.

Bumble Bee Watch data has already been shown to add important information for species conservation status assessments. A next step is comparing user and expert collected data to see if they show the same trends for more species; if this is the case, then community scientists may be capturing an accurate representation of the wild bumble bee community, which are essential baseline data for conservation purposes. Examining accuracy can also lead to increased understanding of participants’ motivations, for example if submissions of a species (and related misidentifications) peak in correlation to federal listing activity, or relatedly, if at-risk species are reported more frequently than are found. There were 45 provinces/territories/states with less than 100 records submitted and reviewed in this data set (Table S1). Promotion of the program in jurisdictions with low participation, particularly in the areas with less than 5 records as of the date of our data export (Alaska, District of Columbia, Rhode Island, South Dakota, West Virginia, and the Yukon) can also help to increase the coverage and value of the program.

## Conclusion

The goal of Bumble Bee Watch is to track and conserve the bumble bees of North America. It has been successful in collecting tens of thousands of species records over broad temporal and geographic scales, including records of at-risk species, and the data has already fed into status assessments and other conservation tools and actions. With an urgency in the lack of local and global pollinator species data available (Potts et al., 2010), projects such as Bumble Bee Watch are filling a time-sensitive gap. For community science programs to produce scientifically rigorous data for conservation policy and management, data must undergo quality assurance protocols. Our work found that for Bumble Bee Watch, the accuracy of user identification varies greatly depending on the species, and expert review is needed for most records, but at-risk species are not always over-reported as compared to common ones.

Work to increase the accuracy of species identification in community science may benefit the users, experts, and project overall. Further investigations into the trends and causes of overall initial accuracy is needed. New educational materials and various checkpoints in the pathways to identification should be developed to assist in identification: this could speed up or potentially even eliminate the need for expert review for at least some species, and help users increase their knowledge and identification skills.

Future research may also focus on examining the connection between government activity and/or media attention (e.g., listing of a species as endangered, regional program spotlights) and increases in community scientist participation. Relatedly, it is also important to determine how best to recruit and maintain the interest of a diverse group of users from across North America, to ensure the Bumble Bee Watch program increases its capacity to capture a detailed picture of our bumble bees.

## Supplemental Information

10.7717/peerj.9412/supp-1 Supplemental Information 1 Tables S1–S6.

10.7717/peerj.9412/supp-2 Supplemental Information 2 An anonymized excerpt of the Bumble Bee Watch database used in the analyses of accuracy. Data were exported from Bumble Bee Watch on July 19, 2019.

10.7717/peerj.9412/supp-3 Supplemental Information 3 Excerpts of the anonymized raw survey responses by users and experts (portion related to perception of ease of identification).

## Additional Information and Declarations

### Data Availability

The following information was supplied regarding data availability: An anonymized excerpt of the Bumble Bee Watch database is available as a Supplemental File. Excerpts of the anonymized raw survey responses by users and experts (portion related to perception of ease of identification) are also available as a Supplemental File.
