# Supplementary material for: Using Bumble Bee Watch to investigate the accuracy and perception of bumble bee (Bombus spp.) identification by community scientists
Source: PeerJ. 2020 Jun 29;8:e9412. doi: 10.7717/peerj.9412 (PMC7331626; doi:10.7717/peerj.9412)
Supplement: Supplemental Information 1 [file peerj-08-9412-s001.pdf]

Supplemental Files for:

Using Bumble Bee Watch to investigate the accuracy and perception of bumble bee (*Bombus* spp.) identification by community scientists

Victoria J. MacPhail[1], Shelby D. Gibson[2], Rich Hatfield[3], Sheila R. Colla[1]

[1] Faculty of Environmental Studies, York University, Toronto, Ontario, Canada
[2] Department of Biology, York University, Toronto, Ontario, Canada
[3] Xerces Society for Invertebrate Conservation, Portland, Oregon, USA

Corresponding Author
Victoria MacPhail[1]
4700 Keele St, Toronto, Ontario, M3J 1P3 Canada
Email address: macphail@yorku.ca

(Original Article Published in PeerJ 2020)

MacPhail et al. 2020. Accuracy of Citizen Scientists on Bumble Bee Watch. Peer J. Supplemental Table S1. The number and relative percent abundance of bumble bee (*Bombus* spp) records, and their expert review status per province, territory, or state, as used in our analyses of user accuracy. Ranks relate the standing of each jurisdiction regarding the relative percent of records submitted from that jurisdiction, with a higher relative abundance yielding a higher rank; tied ranks indicate identical percentages.

Province, State, Territory	# Invalid Records	# Verified Records	Total # Records	Relative Percent Abundance	Rank
Alabama	43	13	56	0.25	30
Alaska	0	2	2	0.01	58
Alberta	63	1077	1140	5.14	6
Arizona	9	3	12	0.05	50
Arkansas	3	3	6	0.03	51
British Columbia	36	474	510	2.30	8
California	167	217	384	1.73	9
Colorado	11	60	71	0.32	26
Connecticut	4	2	6	0.03	51
Delaware	1	14	15	0.07	47
District of Columbia	0	3	3	0.01	58
Florida	28	11	39	0.18	34
Georgia	25	16	41	0.19	32
Idaho	68	2234	2302	10.39	4
Illinois	22	102	124	0.56	17
Indiana	22	32	54	0.24	31
Iowa	8	74	82	0.37	24
Kansas	8	6	14	0.06	48
Kentucky	7	7	14	0.06	48
Louisiana	4	1	5	0.02	56
Maine	6	82	88	0.40	19
Manitoba	17	222	239	1.08	12
Maryland	20	50	70	0.32	26
Massachusetts	19	68	87	0.39	21
Michigan	34	154	188	0.85	15
Minnesota	96	1458	1554	7.01	5
Mississippi	5	2	7	0.03	51
Missouri	16	23	39	0.18	34
Montana	10	29	39	0.18	34
Nebraska	6	33	39	0.18	34
Nevada	13	5	18	0.08	44
New Brunswick	10	179	189	0.85	15
New Hampshire	7	29	36	0.16	38
New Jersey	12	22	34	0.15	39

Province, State, Territory	# Invalid Records	# Verified Records	Total # Records	Relative Percent Abundance	Rank
New Mexico	3	4	7	0.03	51
New York	16	74	90	0.41	18
Newfoundland and Labrador	13	59	72	0.32	26
North Carolina	19	41	60	0.27	29
North Dakota	1	16	17	0.08	44
Northwest Territories	4	2	6	0.03	51
Nova Scotia	19	324	343	1.55	11
Ohio	60	150	210	0.95	13
Oklahoma	4	14	18	0.08	44
Ontario	611	5536	6147	27.74	1
Oregon	190	2347	2537	11.45	3
Pennsylvania	26	60	86	0.39	21
Prince Edward Island	0	43	43	0.19	32
Quebec	20	360	380	1.71	10
Rhode Island	1	3	4	0.02	56
Saskatchewan	15	192	207	0.93	14
South Carolina	15	14	29	0.13	40
South Dakota	1	2	3	0.01	58
Tennessee	11	11	22	0.10	42
Texas	49	28	77	0.35	25
Utah	8	16	24	0.11	41
Vermont	2	82	84	0.38	23
Virginia	26	63	89	0.40	19
Washington	115	3310	3425	15.46	2
West Virginia	1	1	2	0.01	58
Wisconsin	33	616	649	2.93	7
Wyoming	3	16	19	0.09	43
Yukon	0	2	2	0.01	58
Grand Total	2066	20093	22159	100.00	

MacPhail et al. 2020. Accuracy of Citizen Scientists on Bumble Bee Watch. Peer J.
Supplemental Table S2. Results of Chi-Square (χ2) or Fisher's Exact Tests (FET) and Cohen's Kappa values investigating the agreement between user submitted and expert identifications for all records in the Bumble Bee Watch citizen science program, as evaluated on a per species basis. When the Chi-square test assumption of expected values being >0 and 80% of records being >5 were not met, FET were conducted to provide the significance (p) values. No statistic value is provide by the FET in these instances. As there were no verified records of *B. variabilis* despite user-submitted identifications, no Chi-Square or Kappa values could be calculated. The degrees of freedom associated with the Chi-Square and FET statistic was 1 for each species when considered separately and 1681 for all species together. Significance levels for Kappa were <0.0001 except for *B. bohemicus*, which was 0.96. Cohen's Kappa values as per Landis and Koch (1977): < 0: no observer agreement; 0–0.20: slight observer agreement; 0.21-0.40: fair observer agreement; 0.41-0.60: moderate observer agreement; 0.61-0.80: substantial observer agreement; 0.81-1: almost perfect observer agreement.

***Bombus* species**	χ2 or FET test?	Chi-Square (χ2) Value	Sig. Value for χ2 or FET	Kappa Value
affinis	χ2	9,144.64	<0.0001	0.59
appositus	FET	n/a	<0.0001	0.47
auricomus	FET	n/a	<0.0001	0.49
bifarius	χ2	12,117.38	<0.0001	0.74
bimaculatus	χ2	8,479.12	<0.0001	0.62
bohemicus	FET	n/a	1.00	0.00
borealis	FET	n/a	<0.0001	0.69
caliginosus	FET	n/a	<0.0001	0.29
centralis	χ2	10,162.21	<0.0001	0.67
citrinus	FET	n/a	<0.0001	0.44
crotchii	FET	n/a	<0.0001	0.57
cryptarum	FET	n/a	<0.0001	0.54
fervidus	χ2	7,999.26	<0.0001	0.60
flavidus	FET	n/a	<0.0001	0.54
flavifrons	χ2	7,436.08	<0.0001	0.57
franklini	χ2	7,374.49	<0.0001	0.57
fraternus	FET	n/a	<0.0001	0.63
frigidus	FET	n/a	<0.0001	0.37
griseocollis	χ2	8,985.17	<0.0001	0.63
huntii	χ2	12,375.80	<0.0001	0.74
impatiens	χ2	9,755.90	<0.0001	0.65
insularis	FET	n/a	<0.0001	0.32
kirbiellus	FET	n/a	<0.0001	0.04
melanopygus	χ2	6,553.95	<0.0001	0.54
mixtus	χ2	11,561.33	<0.0001	0.71
morrisoni	FET	n/a	<0.0001	0.60

nevadensis	$\chi2$	11,989.26	<0.0001	0.74
occidentalis	FET	n/a	<0.0001	0.71
pensylvanicus	FET	n/a	<0.0001	0.41
perplexus	$\chi2$	9,995.50	<0.0001	0.67
rufocinctus	$\chi2$	4,183.46	<0.0001	0.43
sandersoni	FET	n/a	<0.0001	0.05
sitkensis	FET	n/a	<0.0001	0.42
sp.	n/a	n/a	n/a	n/a
suckleyi	FET	n/a	0.002	0.04
sylvicola	FET	n/a	<0.0001	0.22
ternarius	$\chi2$	12,490.63	<0.0001	0.74
terricola	FET	n/a	<0.0001	0.72
Unknown (Non-*Bombus*)	n/a	n/a	n/a	n/a
vagans	$\chi2$	4,287.25	<0.0001	0.44
vagans, sandersoni or *perplexus*	n/a	n/a	n/a	n/a
vandykei	FET	n/a	<0.0001	0.43
variabilis	n/a	n/a	n/a	n/a
vosnesenskii	$\chi2$	10,642.80	<0.0001	0.69
vosnesenskii or *caliginosus*	FET	n/a	0.017	0.01
**Grand Total**	FET	n/a	<0.0001	0.50

MacPhail et al. 2020. Accuracy of Citizen Scientists on Bumble Bee Watch. Peer J.
Supplemental Table S3. Results of pair-wise Bonferroni-corrected post-hoc tests investigating the relationship between the percent agreement and veracity for each bumble bee species (Bombus spp.) submitted to Bumble Bee Watch as compared to the IUCN Red List value for the species. The IUCN red list status is for each species across its North American range, as per a July 30, 2019 export (IUCN 2019). IUCN ranks are as follows: 1 – Least Concern; 2 – Vulnerable; 3 – Endangered; 4 – Critically Endangered. Species that are considered Data Deficient by the IUCN are not presented here. *Bombus variabilis* and *B. franklini* were removed from analyses involving veracity there were no verified records in the dataset. Marginal mean difference refers to the difference in marginal means between each associated IUCN Red List pair.

		Percent Agreement (total n= 37)			Veracity (total n= 35)		
IUCN Red List	IUCN Red List	Marginal Mean Difference	Std. Error	Sig.	Marginal Mean Difference	Std. Error	Sig.
1	2	18.95	10.230	.384	-15.33	5.802	.049
	3	18.02	4.943	.002	-40.50	4.845	<0.001
	4	46.19	12.669	.002	-44.50	3.078	<0.001
2	1	-18.95	10.230	.384	15.33	5.802	.049
	3	-.93	9.081	1.000	-25.17	6.359	<0.001
	4	27.24	14.783	.392	-29.17	5.142	<0.001
3	1	-18.02	4.943	.002	40.50	4.845	<0.001
	2	.93	9.081	1.000	25.17	6.359	<0.001
	4	28.17	11.761	.100	-4.00	4.031	1.000
4	1	-46.19	12.669	.002	44.50	3.078	<0.001
	2	-27.24	14.783	.392	29.17	5.142	<0.001
	3	-28.17	11.761	.100	4.00	4.031	1.000

MacPhail et al. 2020. Accuracy of Citizen Scientists on Bumble Bee Watch. Peer J. Supplemental Table S4. A comparison of the means and estimates of the marginal means resulting from descriptive statistics and pair-wise Bonferroni-corrected post-hoc tests investigating the relationship between the percent agreement and veracity for each bumble bee species (*Bombus* spp.) submitted to Bumble Bee Watch as compared to the IUCN Red List value for the species. The IUCN red list status is for each species across its North American range, as per a July 30, 2019 export (IUCN 2019). IUCN ranks are as follows: 1 – Least Concern; 2 – Vulnerable; 3 – Endangered; 4 – Critically Endangered. Species that are considered Data Deficient by the IUCN are not presented here. *Bombus variabilis* and *B. franklini* were removed from analyses involving veracity there were no verified records in the dataset.

IUCN Red List	Variable	Standard Mean			Estimated Marginal Means	
		Mean	Std. Deviation	N	Mean	Std. Error
1	Percent Agreement	61.5224	24.5961	25	61.52	4.828
2	Percent Agreement	42.5514	25.81192	7	42.57	9.019
3	Percent Agreement	43.78	2.36174	2	43.50	1.061
4	Percent Agreement	15.1967	24.70269	3	15.33	11.713
1	Veracity	53.9892	14.71108	25	54.00	2.889
2	Veracity	69.34	13.36435	6	69.33	5.031
3	Veracity	94.445	7.85596	2	94.50	3.889
4	Veracity	98.26	2.46073	2	98.50	1.061

MacPhail et al. 2020.Using Bumble Bee Watch to investigate the accuracy of bumble bee (Bombus spp.) identification by community scientists . Peer J. Supplemental Table S5. A comparison of the species names as submitted by users to those species names later assigned by experts. The original user submitted species names are along the first row, and the expert identifications are down the first column. Numbers in each cell relate to the total numbers of records that match both the user-submitted species name in the first row and the expert-reviewed species name (or category) in the first columns of the table.

	**Row: observer-submitted species name**															
**Column: expert-reviewed species name**	affinis	appositus	auricomus	bifarius	bimaculatus	bohemicus	borealis	caliginosus	centralis	citrinus	crotchii	cryptarum	fervidus	flavidus	flavifrons	franklini
affinis	333															
appositus		138		7									6		2	
auricomus			72				2						2			
bifarius		2		985									10	3	9	
bimaculatus	45		5		800	2	2			7			1		2	
bohemicus													2			
borealis	2		3				191						40			
caliginosus								43								
centralis		2		22					291						15	
citrinus	8				1					43			1			
crotchii			1								8					
cryptarum												40	2			
fervidus		2	1	10			12	3	1		1		368	2	1	
flavidus				2									5	57		
flavifrons		9	2	19					35		1		12	1	461	
franklini																
fraternus																
frigidus				1												
griseocollis	64		5	2	36			1		1			3		25	
huntii		3		27					4				2		3	
impatiens	207		41		246	9				16	2		25		12	
insularis		2		20									42	16	4	
kirbiellus																
melanopygus		1	1	35				1	35		1				21	
mixtus		1		20			1	1	4		1		2	3	27	
morrisoni		1											1			
nevadensis	1	1		1									3		4	
occidentalis				3									2			
pensylvanicus			10								1		5			
perplexus					10		1			6			3			
rufocinctus	12	2	9	28	10	2	4	2	9	3			12		13	
sandersoni													3			
sitkensis		4		1									1		14	
suckleyi																
sylvicola		1		3												
ternarius	30			9			3				2		2			
terricola			8										4			
Unknown/Not a bumble bee	34	6	25	13	29	13	4	3	1	16	1		32	2	16	4
vagans	7		1	11	22				1	2			4		26	
vagans, sandersoni or perplexus	19		5		12	1				5			4			
vandykei				7									2			
variabilis																
vosnesenskii		1		2				95			1		28		1	
vosnesenskii or caliginosus								83		1			14	3		1
**Grand Total**	**762**	**176**	**189**	**1228**	**1166**	**27**	**220**	**232**	**381**	**100**	**19**	**40**	**643**	**87**	**656**	**5**

MacPhail et al. 2020.
Table S5 cont'd

	Row: observer-submitted species name															
**Column: expert-reviewed species name**	fraternus	frigidus	griseocollis	huntii	impatiens	insularis	kirbiellus	melanopygus	mixtus	morrisoni	nevadensis	occidentalis	pensylvanicus	perplexus	rufocinctus	sandersoni
affinis			1													
appositus				1			3	2	1	1	2					
auricomus	2					2					1		10			
bifarius				6		8	1	24			1	17			26	
bimaculatus	1		62		131		4				3		2	1	34	13
bohemicus																
borealis						1	5				5	1	5		5	2
caliginosus						1										
centralis		3	2	17			14	22	6						15	
citrinus	1		2		6	1							1	1	1	1
crotchii																
cryptarum							1					26				
fervidus	1				1	1	7	3	1	1	9	8	6		9	1
flavidus					1	3	1	2	4			2			3	
flavifrons		3	19	2			16	17	8	6	9	11	1		19	
franklini																
fraternus	20															
frigidus		14						1	1						1	
griseocollis	3		921		120	1		1		3	23			2	31	5
huntii				446			2	26	1		1				15	
impatiens	11	1	133		2539	5	5				5	1	22	5	33	23
insularis			2		1	75	2		1		5	4			8	
kirbiellus							3									
melanopygus				23		1	7	309	10			8			19	
mixtus		6		1	1		21	16	437			6			24	
morrisoni										32	4				2	
nevadensis			10		6					4	333			1	19	
occidentalis												218			1	
pensylvanicus	1						2				1	3	45			
perplexus			12		5	2	2	1		1	12			98	11	24
rufocinctus		16	9	20	9	3	10	36			1	1	4	2	422	16
sandersoni					1			2								4
sitkensis								1	15			4			3	
suckleyi																
sylvicola				1				4							1	
ternarius		10		7			6	24	2			1	3		132	
terricola			1		1	1		1				5	8			2
Unknown/Not a bumble bee	4	2	70	7	106	15	19	20	4	9	6	28	10	4	52	15
vagans			28		28	2	1	1			34			2	11	4
vagans, sandersoni or perplexus			9		36		1	1					7	2	6	17
vandykei						6	1			2	1				2	
variabilis																
vosnesenskii			1		1	55		1			1	22				
vosnesenskii or caliginosus			1		1	33		2		1		6			1	
**Grand Total**	**44**	**55**	**1283**	**531**	**2994**	**216**	**134**	**517**	**491**	**60**	**457**	**372**	**124**	**118**	**906**	**127**

MacPhail et al. 2020.
Table S5 cont'd

	**Row: observer-submitted species name**											
**Column: expert-reviewed species name**	sitkensis	sp.	suckleyi	sylvicola	ternarius	terricola	vagans	vandykei	variabilis	vosnesenskii	vosnesenskii or caliginosus	**Grand Total**
affinis		11										**345**
appositus		34	1									**198**
auricomus		6			1	7						**105**
bifarius	2	250	14	26	1	1		1				**1387**
bimaculatus		183				2	35		5			**1340**
bohemicus												**2**
borealis		60		4		4						**328**
caliginosus		3						1		12		**60**
centralis		63		3	1							**476**
citrinus		13				4	7		3			**94**
crotchii												**9**
cryptarum	4	31				3						**107**
fervidus		91	6	6		2				9		**563**
flavidus		39	1	4		1						**125**
flavifrons	13	241		5		1	3	4		1		**919**
franklini												**0**
fraternus												**20**
frigidus		2										**20**
griseocollis		229			2		35		7			**1520**
huntii		85	1	32	11							**659**
impatiens		747	1	4	1	3	55		52	1		**4205**
insularis		41	16	1								**240**
kirbiellus												**3**
melanopygus		103	2	19	3							**599**
mixtus	9	144		5		1						**731**
morrisoni		6										**46**
nevadensis		46					13					**442**
occidentalis		15				1				2		**242**
pensylvanicus		19				6						**93**
perplexus		78					31					**297**
rufocinctus		199	4	17	32	6	23		1			**937**
sandersoni		5				2	4					**21**
sitkensis	38	31					1					**113**
suckleyi			1									**1**
sylvicola		12		24								**46**
ternarius		184		10	727	2						**1154**
terricola		49				198	1					**279**
Unknown/Not a bumble bee	1	1333	2	7	11	13	10	1	73	16		**2037**
vagans		107	2			6	239					**539**
vagans, sandersoni or perplexus		102			1	5	59					**292**
vandykei		17	1	1				25		4		**69**
variabilis												**0**
vosnesenskii		200						9		694		**1112**
vosnesenskii or caliginosus		97	1	2				7		129	1	**384**
**Grand Total**	**67**	**4876**	**53**	**170**	**791**	**268**	**516**	**48**	**141**	**868**	**1**	**22159**

MacPhail et al. 2020. Accuracy of Citizen Scientists on Bumble Bee Watch. Peer J. Supplemental Table S6. The number of unique users who submitted records for each expert-reviewed species, and summary statistics related to the number of observations per person and per species. The number of minimum records per user excluded zeros.

Verified Species	# Verified Records	# Unique Users	Mean # Records /User	SE Mean	Max # Records Per User	Min # Records Per User	Relative Percent Max Records Per User
affinis	345	136	2.54	0.36	31	1	8.99
appositus	198	82	2.41	0.29	19	1	9.60
auricomus	105	61	1.72	0.26	13	1	12.38
bifarius	1387	213	6.51	0.89	134	1	9.66
bimaculatus	1340	281	4.77	0.34	77	1	5.75
bohemicus	2	1	2.00		2	2	100.00
borealis	328	180	1.82	0.18	23	1	7.01
caliginosus	60	22	2.73	0.65	11	1	18.33
centralis	476	175	2.72	0.31	31	1	6.51
citrinus	94	54	1.74	0.26	11	1	11.70
crotchii	9	5	1.80	0.58	4	1	44.44
cryptarum	107	70	1.53	0.19	12	1	11.21
fervidus	563	273	2.06	0.16	32	1	5.68
flavidus	125	45	2.78	0.48	16	1	12.80
flavifrons	919	219	4.20	0.55	76	1	8.27
fraternus	20	12	1.67	0.25	5	1	25.00
frigidus	20	12	1.67	0.58	8	1	40.00
griseocollis	1520	268	5.67	0.24	84	1	5.53
huntii	659	267	2.47	0.20	27	1	4.10
impatiens	4205	1447	2.91	0.33	271	1	6.73
insularis	240	87	2.76	0.44	25	1	10.42
kirbiellus	3	3	1.00	0.00	1	1	33.33
melanopygus	599	263	2.28	0.17	33	1	5.51
mixtus	731	217	3.37	0.43	54	1	7.39
morrisoni	46	34	1.35	0.11	3	1	6.52
nevadensis	442	241	1.83	0.14	20	1	4.52
occidentalis	242	96	2.52	0.32	22	1	9.09
pensylvanicus	93	72	1.29	0.11	6	1	6.45
perplexus	297	153	1.94	0.24	28	1	9.43
rufocinctus	937	256	3.66	0.19	32	1	3.42
sandersoni	21	17	1.24	0.14	3	1	14.29
sitkensis	113	40	2.83	0.55	16	1	14.16
suckleyi	1	1	1.00		1	1	100.00
sylvicola	46	15	3.07	1.17	14	1	30.43
ternarius	1154	250	4.62	0.28	100	1	8.67

Verified Species	# Verified Records	# Unique Users	Mean # Records /User	SE Mean	Max # Records Per User	Min # Records Per User	Relative Percent Max Records Per User
terricola	279	136	2.05	0.26	21	1	7.53
Unknown/non -*Bombus*	2037	248	8.21	0.03	20	1	0.98
vagans	539	200	2.70	0.28	28	1	5.19
vagans, sandersoni or *perplexus*	292	187	1.56	0.16	22	1	7.53
vandykei	69	19	3.63	0.76	11	1	15.94
vosnesenskii	1112	243	4.58	0.25	53	1	4.77
vosnesenskii or *caliginosus*	384	241	1.59	0.09	11	1	2.86
totals overall all species combined	22159	4912	4.51	0.27	448	1	2.02
